# Strengthening the One Health Agenda: The Role of Molecular Epidemiology in *Aspergillus* Threat Management

**DOI:** 10.3390/genes9070359

**Published:** 2018-07-19

**Authors:** Eta E. Ashu, Jianping Xu

**Affiliations:** 1 Department of Biology, McMaster University, 1280 Main St. W, Hamilton, Ontario, ON L8S 4K1, Canada; ashue@mcmaster.ca; 2 Public Research Laboratory, Hainan Medical University, Haikou, Hainan 571199, China

**Keywords:** molecular epidemiology, One Health, *Aspergillus fumigatus*, invasive fungal diseases, threat management

## Abstract

The United Nations’ One Health initiative advocates the collaboration of multiple sectors within the global and local health authorities toward the goal of better public health management outcomes. The emerging global health threat posed by *Aspergillus* species is an example of a management challenge that would benefit from the One Health approach. In this paper, we explore the potential role of molecular epidemiology in *Aspergillus* threat management and strengthening of the One Health initiative. Effective management of *Aspergillus* at a public health level requires the development of rapid and accurate diagnostic tools to not only identify the infecting pathogen to species level, but also to the level of individual genotype, including drug susceptibility patterns. While a variety of molecular methods have been developed for *Aspergillus* diagnosis, their use at below-species level in clinical settings has been very limited, especially in resource-poor countries and regions. Here we provide a framework for *Aspergillus* threat management and describe how molecular epidemiology and experimental evolution methods could be used for predicting resistance through drug exposure. Our analyses highlight the need for standardization of loci and methods used for molecular diagnostics, and surveillance across *Aspergillus* species and geographic regions. Such standardization will enable comparisons at national and global levels and through the One Health approach, strengthen *Aspergillus* threat management efforts.

## 1. The Genus *Aspergillus*

Fungal infections affect over a billion people and cause approximately 1.5 million deaths each year worldwide [1]. Regrettably, due to increases in the number of at-risk populations, fungal infections are projected to rise [1,2]. It is estimated that death can be averted in over 80% of fungal disease patients through improved diagnostics, treatment surveillance, and effective antifungal therapies [1]. However, to achieve such success, an inter-disciplinary approach is needed. An emerging example of the inter-disciplinary approach is the One Health initiative. The World Health Organization defines One Health as ‘an approach to designing and implementing programs, polices, legislation, and research in which multiple sectors communicate and work together to achieve better public health outcomes’.

Species in the ascomycete genus *Aspergillus* have emerged as key agents of the fungal infections around the world [3,4,5,6,7,8,9,10,11,12,13,14,15,16,17,18,19]. For example, *Aspergilli* are the leading cause of chronic severe and allergic fungal infections, and the second leading cause of acute invasive fungal infections [1]. The genus *Aspergillus* was first described at the end of the 18th century by a Catholic priest and botanist named Pier Antonio Micheli. Viewing the microscopic spore-bearing structure of *Aspergillus*, Micheli was reminded of a holy water sprinkler—an aspergillum [20,21,22]. Since then, the number of species in genus *Aspergillus* has grown to encompass eight subgenera and over 250 species [23,24]. Of these species, approximately 15% are of known clinical importance [25,26]. DNA sequence-based methods are revealing an increasing number of cryptic species of *Aspergillus* associated with human diseases [27,28,29,30]. For example, surveys carried out in the United States, Brazil, and Spain revealed the percentage of phylogenetically divergent lineages representing cryptic *Aspergilli* species among clinical samples to be between 11–19%, a percentage which is notably higher than those seen in other clinically important filamentous fungi, including those belonging to the orders Mucorales, Microascales, and Hypocreales [31,32,33]. This is particularly important given that, in addition to being pathogenic, up to 40% of these cryptic *Aspergilli* can be resistant to antifungal drugs [30,32]. Of greater importance is the fact that some of these cryptic *Aspergilli* are resistant to multiple antifungals which can exacerbate infections caused by these species. Indeed, fungal infections, including those caused by *Aspergilli*, have become a menace to global public health.

*Aspergilli* cause a wide range of infections, commonly referred to as aspergillosis. Allergic bronchopulmonary, chronic pulmonary, and invasive aspergillosis (IA) are the three most common types of *Aspergillus* infections. Allergic bronchopulmonary and chronic pulmonary aspergillosis results from immune hypersensitivity and scarring due to an *Aspergillus* respiratory tract infection. On the other hand, IA can affect a wider range of body organs belonging to the urinary, digestive, and nervous systems. A significant proportion of *Aspergillus* infections are asymptomatic. However, in patients with symptomatic infections, most symptoms are non-specific, and include low-grade fevers, generalized malaise, wheezing, headaches, and haemoptysis [34,35]. Approximately eight million people world-wide are estimated to have aspergillosis [1]. Invasive aspergillosis is the most lethal type of aspergillosis and is estimated to affect >300,000 people globally every year, with a mortality rate as high as 90% in at-risk populations [1,36]. Allergic bronchopulmonary and chronic pulmonary aspergillosis affect approximately 4.8 and 3 million people annually, respectively [1].

Although aspergillosis cases are predominantly sporadic, outbreaks are not uncommon. Specifically, there have been at least 75 documented aspergillosis outbreaks between January 1966 and December 2015 [37,38,39,40,41,42,43,44,45]. Interestingly, a recent study showed a non-construction-related outbreak that was associated with high airborne spore concentrations in hospital areas with low efficiency air filters [44]. These results highlight the threat posed if environmental spore concentrations reach critical levels within community or home settings. Multiple *Aspergillus* species including *A. fumigatus*, *A. flavus*, *A. terreus*, *A. niger*, *A. glaucus*, *A. oryzae*, and *A. ustus* are known to have caused outbreaks. Among these, *A. fumigatus*, and *A. flavus* are the most frequently identified species [38], and are responsible for approximately 87% of all aspergillosis case reports [26].

Changes in the antifungal susceptibility patterns of these two species have further increased the threat posed by *Aspergilli.* Since its emergence in 1997, resistance to triazole in *A. fumigatus* has steadily increased and is a current global health menace [46,47]. Furthermore, in *A. fumigatus,* there are emerging reports of increased resistance to polyenes such as amphotericin B (AMB), an antifungal to which very little resistance has been reported thus far [48,49]. For example, a recent study carried out in Brazil showed that 27% of a clinical sample of *A. fumigatus* isolates was resistant to AMB (minimum inhibitory concentration (MIC) > 2 mg/L) [48]. Similarly, our group also very recently found that 96% of a combined environmental and clinical sample from Hamilton, Canada was resistant to amphotericin. The recent emergence of voriconazole (VRC) resistance in *A. flavus* will likely cause significant problems in the management of aspergillosis caused by *A. flavus.* Triazoles, especially VRC, are first-line drugs used in the treatment of aspergillosis [50,51,52,53]. Although not yet reported, multi-drug resistant aspergillosis outbreaks similar to those caused by *Candida auris* and *Acinetobacter baumannii* will likely emerge in the near future [54,55,56,57,58].

With the increasing number of clinically important *Aspergillus* species and the changing antifungal susceptibility patterns of key *Aspergilli* such *A. fumigatus*, and *A. flavus,* there is a pressing need to develop novel and effective *Aspergillus* threat management strategies. Below, we propose a framework that can be used in *Aspergillus* threat management. When put in context, this framework can also be used in the management of *Candida* and other clinically relevant fungi. We encourage essential stakeholders to engage in discussions aimed at *Aspergillus* threat management.

## 2. Molecular Epidemiology in *Aspergillus* Threat Management

**Figure 1:**
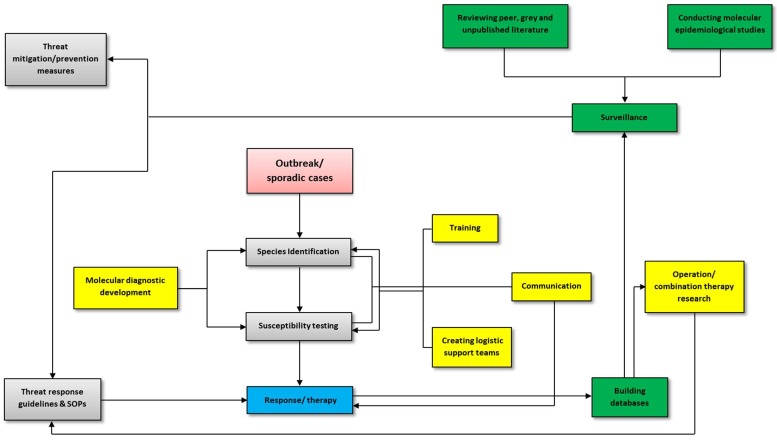
Framework for the management of *Aspergillus* outbreaks and critically important sporadic cases. Activities pertaining to threat preparedness are highlighted in yellow, while those pertaining to prevention are in green. Training within the context of this review refers to training laboratory technicians to perform rapid and accurate diagnosis of *Aspergilli*, including resistant strains and cryptic species. It also entails cross-training other laboratory technicians to carry out the designated emergency technician’s routine duties. Communication here covers having a standardized plan to act as quickly as possible in relaying information on species and susceptibility diagnosis to the appropriate people, in order to ensure an adequate response and the safety of groups or persons at risk. Logistic support teams will be responsible for ensuring diagnostic supplies, admission documentation, travel arrangements, quality control, and all components essential for rapid and accurate diagnosis are in place in the event of an *Aspergillus* threat. SOP: standard operating procedure.

A variety of host, pathogen, host-pathogen interaction, and environmental factors have been identified as contributors to the increased threat caused by *Aspergilli*. Of interest among pathogen-related factors is the recent global rise in resistance to antifungal drugs. Triazole resistance in *A. fumigatus* has now been reported in every continent but Antarctica [47]. Generally speaking, pathogen threat management has three interdependent components: preparedness, response, and prevention. In Figure 1, we suggest a non-exclusive framework that could be used in the management of *Aspergillus* threats, including those caused by resistant strains. This review however only focuses on the molecular epidemiology components of preparedness and prevention; specifically, on molecular diagnostics and surveillance (Figure 1).

### 2.1. The Usefulness of Molecular Epidemiology in Aspergillus Surveillance

In epidemiology, surveillance is defined as the collection and analysis of data necessary to develop, implement, and evaluate preventative health measures. Over the last two decades, molecular epidemiology has emerged as a very important tool in the surveillance of diverse human pathogens [59,60]. This burgeoning branch of epidemiology merges traditional epidemiology and molecular biology in order to better characterize virulence, pathogen transmission patterns, and outbreak incidence. In molecular epidemiology, marker genes are used to elucidate the genotypes and the relationship between strains and populations. In addition, some of these marker genes are becoming indispensable for understanding virulence determinants and the distribution of aspergillosis.

Over the years, a wide range of molecular markers has been used to study the molecular epidemiology of *Aspergilli.* These markers include multilocus sequences, microsatellites, PCR-restriction fragment length polymorphisms, Southern hybridization of restriction enzyme-digested DNA, randomly amplified polymorphic DNA, and mating type genes [61,62]. For instance, using microsatellite markers, Guinea and colleagues investigated an aspergillosis outbreak in a major heart surgery unit of a hospital in Spain and showed that such markers were a valuable tool in IA outbreak source investigation [41]. It is however, important to note that aspergillosis outbreaks most often do not have a single source and can consist of a series of unrelated events. As such, pinpointing the source of aspergillosis outbreaks can be difficult [63]. In contrast, molecular markers have been used with more success in determining the sources of infections in non-outbreak cases. For example, a recent study highlighted that the home environment can be an important source of infection for isolated cases of triazole-resistant *A. fumigatus* [64].

In addition to its value in infection source investigation, molecular epidemiology can be used to track *Aspergilli* transmission patterns. For example, using microsatellite markers, a recent global study showed that resistant populations of *A. fumigatus* are significantly differentiated geographically [65]. This result suggests that it may be possible to track triazole resistant *A. fumigatus* strains across national and regional borders. However, significant caution should be applied here. Compared to the large global population of *Aspergilli*, relatively few isolates and genotypes have been analyzed to date, and our current understanding of the molecular variation between and within these populations may not be representative of the true global diversity. In regard to tracking *Aspergilli*, geographic sub-structuring can vary by country and region, hence tracking transmission patterns of clinically relevant *Aspergilli* within or between certain countries might prove to be easier than in others [39,66]. For example, little to no geographic population structuring has been reported in *A. fumigatus* samples from India and Netherlands, whereas Cameroonian *A. fumigatus* samples show significant evidence for geographic sub-structuring [66]. As a result, tracking clinically relevant *A. fumigatus* strains would be a more feasible task in Cameroon than it would be in India or Netherlands.

Aside from transmission pattern tracking and outbreak incidence investigation, molecular markers can also be used in virulence and antifungal drug-resistance characterization. Mating type loci genotyping has been successfully used to characterize the degree of disease severity caused by *A. fumigatus* strains [67,68,69], making it possible for these markers to be used in mapping the distribution of hyper-virulent strains. Similarly, certain mutations in the *cyp51A* gene are known to cause triazole drug resistance. Being able to map the distribution of hyper-virulent and drug resistant *Aspergilli* is essential for emergency preparedness and infection control efforts.

With reference to the wide range of markers used in genotyping *Aspergilli*, whilst molecular epidemiology holds promise for *Aspergilli* surveillance, there are still two pertinent issues that need to be addressed in order for the surveillance data to be fully implemented at the global scale. Firstly, we need a consensus on which markers should be used for the surveillance of *Aspergilli* of public health importance. Secondly, we need to establish and curate a reliable genotype database on the consensus gene markers to which similar information on new isolates can be added and compared. At present, there is a multilocus sequence type database for *A. fumigatus* based on seven loci (https://pubmlst.org/afumigatus/) [70]. However, such a database is not available for other *Aspergillus* species. In addition, while the current multilocus sequence typing (MLST) method for *A. fumigatus* is highly reproducible and can distinguish strains at the genus and species levels, it has a relatively low discriminatory power among strains within the same species [70]. Instead, due to their high polymorphism and greater discrimination power, a group of nine microsatellite markers is more commonly used for genotyping *A. fumigatus*, making the MLST database of *A. fumigatus* of limited use in epidemiological and population genetic investigations [71]. However, a shared database for strain genotypes based on the microsatellite markers is not available. In the short/medium term, we recommend that future work should use the seven consensus MLST loci for genotyping all *Aspergilli* strains, and for *A. fumigatus*, the additional nine microsatellite loci should be used for genotyping. Furthermore, a publicly available database should be set up to include both types of genetic data. Ultimately, with increasing availability and affordability of next-generation sequencing, whole-genome sequencing (WGS) should be considered for epidemiological monitoring for the long term. Data from WGS will not only be highly discriminatory and commonly archived, but also can help identify genetic polymorphisms associated with virulence and antifungal drug susceptibility. Such data will significantly strengthen national and global *Aspergillus* threat management efforts.

**Figure 2:**
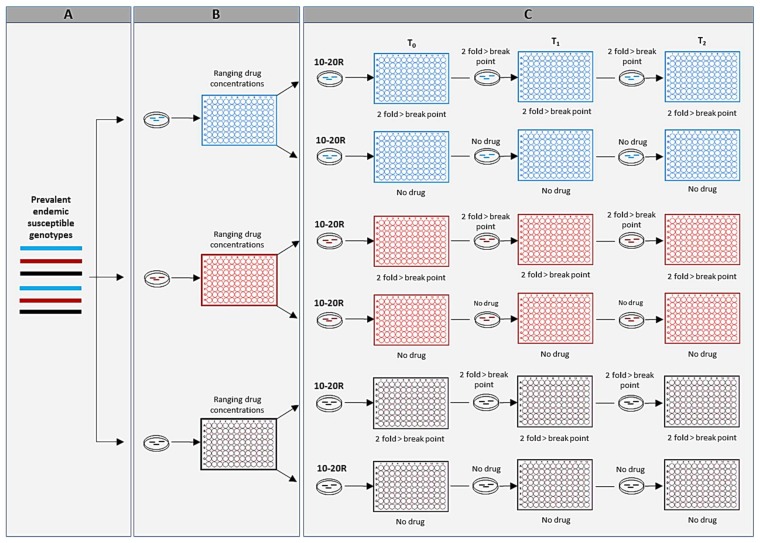
Experimental design to predict resistance through drug exposure in susceptible genotypes. The procedures depicted in panels A and B entail identifying prevalent endemic susceptible genotypes and determining their minimum inhibitory concentrations (MICs) to specific antifungal drugs. All genotypes used for the evolutionary experiment in panel C should have similar starting MICs, preferably very low. The evolutionary experiment consists of continuously exposing 10–20 replicates (R) of the selected genotypes to an antifungal drug as described in the Clinical & Laboratory Standards Institute (CLSI) protocol while including a no-drug control group [75]. At the end of 48 h, MICs are recorded, and new petri dishes are inoculated with microtiter plate content from the previous round of selection. The steps are repeated until genotypes reach resistance break point or epidemiological cutoff values.

Despite the rather successful use of molecular epidemiology in characterizing hyper-virulence, pathogen transmission patterns, and outbreak incidences of *Aspergilli*, very little has been done in leveraging molecular epidemiological methods in predicting drug resistance in endemic wild-type genotypes. For example, wild-type genotypes that are strongly associated with the emergence of resistance have been shown in the human immunodeficiency virus type 1 (HIV-1) [72]. This is particularly important, as infections with wild-type genotypes with higher propensities to become drug resistant could result in inappropriate antifungal therapy, and ultimately lead to treatment failure. In Figure 2, we suggest an in-vitro experimental evolutionary model that can be used in predicting drug resistance likelihood through drug exposure. The above experiment is aimed at measuring two primary outcomes: the likelihood of becoming resistant and the time it takes to attain resistance breakpoint or epidemiological cutoff (resistance time). Data from such experiments can be used to develop statistical learning models that can predict the likelihood of resistance and resistance time in a matter of minutes on a laptop. For example, supposing that acquiring resistance is through a stochastic process related to spontaneous mutation(s), then the likelihood of a genotype becoming resistant can be obtained using the following function *L(G/O)* = *P(O/G)*, where *O* is the observed outcome, *G* is the parameter set (genotype) that define the stochastic process, *L* is likelihood, and *P* is probability. Information on a wild-type genotype’s propensity to become resistant and resistance time can be very useful in determining a treatment course and formulating disease control strategies. Similarly, given the role of sexual reproduction in the emergence of genotypes of clinical importance [63], developing tools capable of predicting highly fit and super mating genotypes is critical, as such genotypes can easily spread resistance genes through sexual reproduction and rapidly expanding their distribution across geographic areas. Indeed, a highly fit multi-triazole resistant *A. fumigatus* genotype which very likely acquired resistance through sexual reproduction has expanded across thousands of kilometers in India [73,74].

In conclusion, from a practical perspective, researchers involved in *Aspergillus* surveillance should monitor the following: (i) genetic structure of the local, regional, national, and global populations of human pathogenic *Aspergillus*; (ii) the distribution and overall prevalence of hyper-virulent strains; (iii) the distribution and overall prevalence of antifungal drug resistant strains including those with a higher propensity to become resistant; and (iv) the transmission patterns of the latter two categories of genotypes. Furthermore, given current increases in non*-fumigatus* aspergillosis, significant attention should be paid to monitoring non-*fumigatus Aspergilli*, especially cryptic species.

### 2.2. The Usefulness of Molecular Epidemiology in Threat Preparedness

Recent increases in globalization have led to more frequent movement of goods and people than ever before in human history. The constant movement of people and goods across geographic scales has significant implications for the spread of clinically important pathogens like *Aspergilli*. Indeed, both anthropogenic and non-anthropogenic activities have impacted the spread of clinically important *Aspergilli* across geographic boarders [65,76]. Such impacts highlight the need for preparedness even in countries with low aspergillosis incidences. There is an age-old adage that says “Luck favors the prepared mind”. Threat preparedness in the context of this review implies being able to effectively anticipate and take the right steps to manage threats caused by *Aspergilli.* Among other things, this entails being able to accurately and rapidly diagnose *Aspergilli*, especially drug-resistant *Aspergilli*. Thus far, multiple diagnostic methods—including electrospray ionization and matrix-assisted laser desorption ionization mass spectrometry, nucleic acid sequence-based amplification, polymerase chain reactions (real-time, multiplex and nested), microsphere-based Luminex, loop-mediated isothermal amplification, and enzyme linked immunosorbent assays—have been used to identify *Aspergilli* in a wide range of specimens including whole blood, serum, plasma, bronchoalveolar lavages, and exhale breath condensate [77,78,79,80,81]. These methods, including their respective advantages and disadvantages, have been extensively reviewed and are not discussed in detail here. Instead, our focus is on providing suggestions necessary for leveraging molecular diagnostics in preparedness against the threat caused by *Aspergilli.*

Specifically, we would like to highlight two critical issues that need to be addressed by many countries in their efforts to manage the threat caused by *Aspergilli*. Firstly, we note that more research needs to be done in developing rapid diagnostic molecular markers for clinically important non-*fumigatus Aspergilli*, including the divergent lineages/cryptic species. This is particularly important because although some of these species represent only a small proportion of clinically diagnosed *Aspergilli*, they form significant proportions of drug-resistant *Aspergilli*. For example, although *A. lentulus* accounted for only ~3% (3/86) of a set of 86 *Aspergillus* isolates obtained from Italy and Netherlands, they represented approximately 21% (3/14) of all (≥ 2 mg/L) voriconazole-resistant strains [82]. Another important example to note is *A. calidoustus*, an emerging pathogen in lung transplants which can also colonize water distribution systems, including those in health care settings [83,84]. Thus, the need for accurate and rapid diagnosis of non-*fumigatus Aspergilli* and cryptic species is becoming increasingly important.

Secondly, we highlight the need for standardization of molecular diagnostic procedures and markers at country and regional levels. Generally speaking, of all molecular diagnostic platforms, PCR platforms seem to show the best potential as they are able to identify *Aspergilli* to the species level while also being able to help identify antifungal susceptibility patterns. Thus far, plasma samples have resulted in the highest sensitivity (91%), while whole blood produced the highest specificity (96%) [85]. The highest diagnostic sensitivity and specificity in bronchoalveolar lavages samples thus far were 91% and 92%, respectively [86]. In cerebrospinal fluid, an *Aspergillus*-specific nested PCR assay showed sensitivity and specificity values of 100% and 93%, respectively [87]. Despite the great potential of PCR platforms, the specificity and sensitivity of PCR can be notably affected by two key factors: (i) method of DNA extraction; and (ii) specimen type. It was recently shown that factors including bead beating, white cell lysis, elution, and specimen volume can notably affect the quality and quantity of extracted DNA, and consequently PCR sensitivity [88]. In spite of the significant progress made by the European *Aspergillus* PCR initiative in quelling variation associated with DNA extraction from whole blood [89], the blood fraction best suited for PCR assays is still disputed. Furthermore, PCR performance is still to be evaluated in a wide range of patients, with other types of immunosuppressive conditions as most specimens used for PCR assays this far were performed on samples from hematology-oncology patients. Similarly, the differences in bronchoalveolar lavage procedures between patients and medical centers can affect PCR interpretation [90]. Indeed, there is room for improvements in all molecular diagnostic platforms.

Regardless of these shortfalls, molecular diagnostic platforms such as PCR can still be leveraged in the management of *Aspergilli* threats in two key ways. Firstly, molecular diagnostic platforms can be used in combinations to obtain optimal results. For example, a recent study showed that combining a lateral flow device with PCR yielded 100% diagnostic sensitivity and specificity [91]. Similarly, an earlier diagnosis and a lower incidence of IA were associated with a combination of galactomannan (GM) and PCR-based *Aspergillus* detection [92]. Secondly, PCR and similar molecular diagnostic platforms can be preemptively used in the surveillance of high risk patients in order to provide rapid usable results if needed. For instance, an improved 30-day survival rate was observed in a group that received PCR-guided prophylactic treatment compared to that for those treated on the basis of symptoms alone [93]. Similarly, combined surveillance of serum GM and PCR was shown to help decrease the incidence of IA in at-risk populations [92]. Surveillance of at-risk populations, health care facilities and adjacent environmental areas using molecular diagnostic platforms depicts how interdependent components of pathogen threat management can be. However, combining different platforms for diagnosis, prevention, and treatment requires standardization of procedures across different immunosuppressed populations and the mastery of diverse mycological procedures. Similarly, routine molecular diagnostic surveillance in high risk populations will require a significant amount of human resources. In the context of *Aspergillus* threat management, more specifically preparedness, standard operating procedures for the rapid and accurate diagnosis of both common and uncommon clinically important *Aspergilli* still need to be developed and adopted in many countries. Furthermore, the necessary human resources, cost effectiveness, and research capacity required to achieve the latter should be considered in most clinical microbiology laboratories.

Within individual *Aspergillus* species, having a good understanding of the molecular epidemiology is vital in formulating integrative molecular diagnostic plans. For instance, given that almost all multi-triazole resistant *A. fumigatus* strains in India belonged to a single microsatellite genotype (14/20/9/31/9/10/8/10/28) [74], screening for this genotype in addition to the common *A. fumigatus* species-level gene markers discussed here could diagnose an *A. fumigatus* isolate to a below-species level, including whether it is multi-triazole resistant. Such a plan could also be used in other geographic areas if one or a few genotypes dominate the drug-resistant population.

## 3. Conclusions

In this paper, we highlight the importance of surveillance and molecular diagnostics in *Aspergillus* threat preparedness and prevention. Given how interconnected that the world has become, standardization of loci and methods used for molecular diagnostics and surveillance is critically important for managing the current global *Aspergillus* threat. Although not discussed in detail here, combination therapy recommendations and vaccine research are two other key *Aspergillus* threat preparedness and prevention components worth mentioning.

Effectively managing the current threat caused by *Aspergilli* will require the significant incorporation of the molecular epidemiology component into *Aspergillus* threat preparedness and prevention. From a public health policy perspective, incorporation of genetic information into *Aspergillus* threat management is still a relatively new concept. For example, the European Organization for Research and Treatment of Cancer/Invasive Fungal Infections Cooperative Group and the National Institute of Allergy and Infectious Diseases Mycoses Study Group (EORTC/MSG) only recently suggested the inclusion of molecular diagnostics for the case definition of IA [94]. However, the recommended diagnosis can only identify the infecting pathogen to species level. A recent study identified that different genetic populations within *A. fumigatus* have different rates of triazole resistance [65]. Thus, identifying infecting pathogens to the genotype level will have significant treatment value. Furthermore, molecular markers targeting drug resistance mutations are being developed. The adoption by clinical microbiology labs around the globe of these fine-scale molecular methods will lead to better-targeted treatments and improved patient outcome at the population level.

## References

[1] Bongomin F., Gago S., Oladele R.O., Denning D.W. (2017). Global and multi-national prevalence of fungal diseases—Estimate precision. J. Fungi.

[2] Vallabhaneni S., Mody R.K., Walker T., Chiller T. (2016). The global burden of fungal diseases. Infect. Dis. Clin..

[3] Pegorie M., Denning D.W., Welfare W. (2017). Estimating the burden of invasive and serious fungal disease in the United Kingdom. J. Infect..

[4] Gamaletsou M.N., Drogari-Apiranthitou M., Denning D.W., Sipsas N.V. (2016). An estimate of the burden of serious fungal diseases in Greece. Eur. J. Clin. Microbiol. Infect. Dis..

[5] Gangneux J.-P., Bougnoux M.-E., Hennequin C., Godet C., Chandenier J., Denning D.W., Dupont B. (2016). LIFE program, the Société française de mycologie médicale SFMM-study group. An estimation of burden of serious fungal infections in France. J. Mycol. Med..

[6] Oladele R.O., Denning D.W. (2014). Burden of serious fungal infection in Nigeria. West Afr. J. Med..

[7] Ben R., Denning D.W. (2015). Estimating the burden of fungal diseases in Israel. Isr. Med. Assoc. J. IMAJ.

[8] Taj-Aldeen S.J., Chandra P., Denning D.W. (2015). Burden of fungal infections in Qatar. Mycoses.

[9] Lagrou K., Maertens J., Van Even E., Denning D.W. (2015). Burden of serious fungal infections in Belgium. Mycoses.

[10] Osmanov A., Denning D.W. (2015). Burden of serious fungal infections in Ukraine. Mycoses.

[11] Mortensen K.L., Denning D.W., Arendrup M.C. (2015). The burden of fungal disease in Denmark. Mycoses.

[12] Khwakhali U.S., Denning D.W. (2015). Burden of serious fungal infections in Nepal. Mycoses.

[13] Badiane A.S., Ndiaye D., Denning D.W. (2015). Burden of fungal infections in Senegal. Mycoses.

[14] Klimko N., Kozlova Y., Khostelidi S., Shadrivova O., Borzova Y., Burygina E., Vasilieva N., Denning D.W. (2015). The burden of serious fungal diseases in Russia. Mycoses.

[15] Faini D., Maokola W., Furrer H., Hatz C., Battegay M., Tanner M., Denning D.W., Letang E. (2015). Burden of serious fungal infections in Tanzania. Mycoses.

[16] Sinkó J., Sulyok M., Denning D.W. (2015). Burden of serious fungal diseases in Hungary. Mycoses.

[17] Mandengue C.E., Denning D.W. (2018). The burden of serious fungal infections in Cameroon. J. Fungi.

[18] Guto J.A., Bii C.C., Denning D.W. (2016). Estimated burden of fungal infections in Kenya. J. Infect. Dev. Ctries..

[19] Corzo-León D.E., Armstrong-James D., Denning D.W. (2015). Burden of serious fungal infections in Mexico. Mycoses.

[20] George Agrios (2005). Plant Pathology.

[21] Bennett J.W., Katsuya G., Masayuki M. (2010). An overview of the genus *Aspergillus*. Aspergillus: Molecular Biology and Genomics.

[22] Ashu E., Forsythe A., Vogan A., Xu J., Didier M., Ramesh R. (2016). Filamentous Fungi in Fermented Foods. Fermented Foods, Part I: Biochemistry and Biotechnology.

[23] Dyer P.S., O’Gorman C.M. (2011). A fungal sexual revolution: *Aspergillus* and *Penicillium* show the way. Curr. Opin. Microbiol..

[24] Geiser D.M., Klich M.A., Frisvad J.C., Peterson S.W., Varga J., Samson R.A. (2007). The current status of species recognition and identification in *Aspergillus*. Stud. Mycol..

[25] Sugui J.A., Kwon-Chung K.J., Juvvadi P.R., Latgé J.-P., Steinbach W.J. (2015). *Aspergillus fumigatus* and related species. Cold Spring Harb. Perspect. Med..

[26] Guarro J., Xavier M.O., Severo L.C., Alessandro C.P. (2009). Differences and similarities amongst pathogenic *Aspergillus* species. Aspergillosis: From Diagnosis to Prevention.

[27] Varga J., Houbraken J., Van Der Lee H.A.L., Verweij P.E., Samson R.A. (2008). *Aspergillus calidoustus* sp. nov., causative agent of human infections previously assigned to *Aspergillus ustus*. Eukaryot. Cell.

[28] Balajee S.A., Gribskov J.L., Hanley E., Nickle D., Marr K.A. (2005). *Aspergillus lentulus* sp. nov., a new sibling species of *A. fumigatus*. Eukaryot. Cell.

[29] Gautier M., Normand A.-C., Ranque S. (2016). Previously unknown species of *Aspergillus*. Clin. Microbiol. Infect..

[30] Alastruey-Izquierdo A., Alcazar-Fuoli L., Cuenca-Estrella M. (2014). Antifungal susceptibility profile of cryptic species of *Aspergillus*. Mycopathologia.

[31] Balajee S.A., Kano R., Baddley J.W., Moser S.A., Marr K.A., Alexander B.D., Andes D., Kontoyiannis D.P., Perrone G., Peterson S. (2009). Molecular identification of *Aspergillus* species collected for the transplant-associated infection surveillance network. J. Clin. Microbiol..

[32] Alastruey-Izquierdo A., Mellado E., Peláez T., Pemán J., Zapico S., Alvarez M., Rodríguez-Tudela J.L., Cuenca-Estrella M., Group F.S. (2013). Population-based survey of filamentous fungi and antifungal resistance in Spain (FILPOP Study). Antimicrob. Agents Chemother..

[33] Negri C.E., Gonçalves S.S., Xafranski H., Bergamasco M.D., Aquino V.R., Castro P.T.O., Colombo A.L. (2014). Cryptic and rare *Aspergillus* species in Brazil: Prevalence in clinical samples and in vitro susceptibility to triazoles. J. Clin. Microbiol..

[34] Maturu V.N., Agarwal R. (2015). Itraconazole in chronic pulmonary aspergillosis: In whom, for how long, and at what dose?. Lung India Off. Organ Indian Chest Soc..

[35] Agarwal R. (2009). Allergic bronchopulmonary aspergillosis. Chest.

[36] Dagenais T.R.T., Keller N.P. (2009). Pathogenesis of *Aspergillus fumigatus* in invasive aspergillosis. Clin. Microbiol. Rev..

[37] Vonberg R.-P., Gastmeier P. (2006). Nosocomial aspergillosis in outbreak settings. J. Hosp. Infect..

[38] Weber D.J., Peppercorn A., Miller M.B., Sickbert-Benett E., Rutala W.A. (2009). Preventing healthcare-associated *Aspergillus* infections: Review of recent CDC/HICPAC recommendations. Med. Mycol..

[39] Balajee S.A., Tay S.T., Lasker B.A., Hurst S.F., Rooney A.P. (2007). Characterization of a novel gene for strain typing reveals substructuring of *Aspergillus fumigatus* across North America. Eukaryot. Cell.

[40] Chang C.C., Cheng A.C., Devitt B., Hughes A.J., Campbell P., Styles K., Low J., Athan E. (2008). Successful control of an outbreak of invasive aspergillosis in a regional haematology unit during hospital construction works. J. Hosp. Infect..

[41] Guinea J., García de Viedma D., Peláez T., Escribano P., Muñoz P., Meis J.F., Klaassen C.H.W., Bouza E. (2011). Molecular epidemiology of *Aspergillus fumigatus*: An in-depth genotypic analysis of isolates involved in an outbreak of invasive aspergillosis. J. Clin. Microbiol..

[42] Peláez T., Muñoz P., Guinea J., Valerio M., Giannella M., Klaassen C.H.W., Bouza E. (2012). Outbreak of invasive aspergillosis after major heart surgery caused by spores in the air of the intensive care unit. Clin. Infect. Dis..

[43] Pettit A.C., Kropski J.A., Castilho J.L., Schmitz J.E., Rauch C.A., Mobley B.C., Wang X.J., Spires S.S., Pugh M.E. (2012). The index case for the fungal meningitis outbreak in the United States. N. Engl. J. Med..

[44] Vena A., Muñoz P., Pelaez T., Guinea J., Valerio M., Bouza E. (2015). Non-construction related *Aspergillus* outbreak in non-hematological patients related to high concentrations of airborne spores in non-HEPA filtered areas. Open Forum Infect. Dis..

[45] Kabbani D., Goldraich L., Ross H., Rotstein C., Husain S. (2017). Outbreak of invasive aspergillosis in heart transplant recipients: The role of screening computed tomography scans in asymptomatic patients and universal antifungal prophylaxis. Transpl. Infect. Dis..

[46] Rivero-Menendez O., Alastruey-Izquierdo A., Mellado E., Cuenca-Estrella M. (2016). Triazole resistance in *Aspergillus* spp.: A worldwide problem?. J. Fungi.

[47] Garcia-Rubio R., Cuenca-Estrella M., Mellado E. (2017). Triazole resistance in *Aspergillus* species: An emerging problem. Drugs.

[48] Reichert-Lima F., Lyra L., Pontes L., Moretti M.L., Pham C.D., Lockhart S.R., Schreiber A.Z. (2018). Surveillance for azoles resistance in *Aspergillus* spp. highlights a high number of amphotericin B-resistant isolates. Mycoses.

[49] Ashu E., Korfanty G., Samarasinghe H., Pum N., Man Y., Yamamura D., Xu J. (2018). Widespread presence of amphotericin B resistant *Aspergillus fumigatus* in Hamilton, Canada. Infect. Drug Resist..

[50] Paul R.A., Rudramurthy S.M., Meis J.F., Mouton J.W., Chakrabarti A. (2015). A novel Y319H substitution in CYP51C associated with azole resistance in *Aspergillus flavus*. Antimicrob. Agents Chemother..

[51] Liu W., Sun Y., Chen W., Liu W., Wan Z., Bu D., Li R. (2012). The T788G mutation in the *CYP51C* gene confers voriconazole resistance in *Aspergillus flavus* causing aspergillosis. Antimicrob. Agents Chemother..

[52] Sharma C., Kumar R., Kumar N., Masih A., Gupta D., Chowdhary A. (2018). Investigation of multiple resistance mechanisms in voriconazole-resistant *Aspergillus flavus* clinical isolates from a chest hospital surveillance in Delhi, India. Antimicrob. Agents Chemother..

[53] Nami S., Baradaran B., Mansoori B., Kordbacheh P., Rezaie S., Falahati M., Mohamed Khosroshahi L., Safara M., Zaini F. (2017). The utilization of RNA silencing technology to mitigate the voriconazole resistance of *Aspergillus Flavus*; lipofectamine-based delivery. Adv. Pharm. Bull..

[54] Chowdhary A., Sharma C., Meis J.F. (2017). *Candida auris*: A rapidly emerging cause of hospital-acquired multidrug-resistant fungal infections globally. PLoS Pathog..

[55] Sarma S., Upadhyay S. (2017). Current perspective on emergence, diagnosis and drug resistance in *Candida auris*. Infect. Drug Resist..

[56] Dettori M., Piana A., Deriu M.G., Lo Curto P., Cossu A., Musumeci R., Cocuzza C., Astone V., Contu M.A., Sotgiu G. (2014). Outbreak of multidrug-resistant *Acinetobacter baumannii* in an intensive care unit. New Microbiol..

[57] Ghaith D.M., Zafer M.M., Al-Agamy M.H., Alyamani E.J., Booq R.Y., Almoazzamy O. (2017). The emergence of a novel sequence type of MDR *Acinetobacter baumannii* from the intensive care unit of an Egyptian tertiary care hospital. Ann. Clin. Microbiol. Antimicrob..

[58] Zarrilli R., Casillo R., Di Popolo A., Tripodi M.-F., Bagattini M., Cuccurullo S., Crivaro V., Ragone E., Mattei A., Galdieri N. (2007). Molecular epidemiology of a clonal outbreak of multidrug-resistant *Acinetobacter baumannii* in a university hospital in Italy. Clin. Microbiol. Infect..

[59] Eybpoosh S., Haghdoost A.A., Mostafavi E., Bahrampour A., Azadmanesh K., Zolala F. (2017). Molecular epidemiology of infectious diseases. Electron. Phys..

[60] Villari P., Iacuzio L., Torre I., Scarcella A. (1998). Molecular epidemiology as an effective tool in the surveillance of infections in the neonatal intensive care unit. J. Infect..

[61] De Valk H.A., Klaassen C.H.W., Meis J.F.G.M. (2008). Molecular typing of *Aspergillus* species. Mycoses.

[62] Varga J. (2006). Molecular typing of *Aspergilli*: Recent developments and outcomes. Med. Mycol..

[63] Ashu E.E., Xu J. (2015). The roles of sexual and asexual reproduction in the origin and dissemination of strains causing fungal infectious disease outbreaks. Infect. Genet. Evol..

[64] Lavergne R.-A., Chouaki T., Hagen F., Toublanc B., Dupont H., Jounieaux V., Meis J.F., Morio F., Le Pape P. (2017). Home environment as a source of life-threatening azole-resistant *Aspergillus fumigatus* in immunocompromised patients. Clin. Infect. Dis..

[65] Ashu E.E., Hagen F., Chowdhary A., Meis J.F., Xu J. (2017). Global population genetic analysis of *Aspergillus fumigatus*. MSphere.

[66] Ashu E.E., Korfanty G.A., Xu J. (2017). Evidence of unique genetic diversity in *Aspergillus fumigatus* isolates from Cameroon. Mycoses.

[67] Alvarez-Perez S., Blanco J.L., Alba P., Garcia M.E. (2010). Mating type and invasiveness are significantly associated in *Aspergillus fumigatus*. Med. Mycol..

[68] Cheema M.S., Christians J.K. (2011). Virulence in an insect model differs between mating types in *Aspergillus fumigatus*. Med. Mycol..

[69] Monteiro M.C., Garcia-Rubio R., Alcazar-Fuoli L., Peláez T., Mellado E. (2018). Could the determination of *Aspergillus fumigatus* mating type have prognostic value in invasive aspergillosis?. Mycoses.

[70] Bain J.M., Tavanti A., Davidson A.D., Jacobsen M.D., Shaw D., Gow N.A., Odds F.C. (2007). Multilocus sequence typing of the pathogenic fungus *Aspergillus fumigatus*. J. Clin. Microbiol..

[71] De Valk H.A., Meis J.F.G.M., Curfs I.M., Muehlethaler K., Mouton J.W., Klaassen C.H.W. (2005). Use of a novel panel of nine short tandem repeats for exact and high-resolution fingerprinting of *Aspergillus fumigatus* isolates. J. Clin. Microbiol..

[72] García-Lerma J.G., Nidtha S., Blumoff K., Weinstock H., Heneine W. (2001). Increased ability for selection of zidovudine resistance in a distinct class of wild-type HIV-1 from drug-naive persons. Proc. Natl. Acad. Sci. USA.

[73] Abdolrasouli A., Rhodes J., Beale M.A., Hagen F., Rogers T.R., Chowdhary A., Meis J.F., Armstrong-James D., Fisher M.C. (2015). Genomic context of azole resistance mutations in *Aspergillus fumigatus* determined using whole-genome sequencing. MBio.

[74] Chang H., Ashu E., Sharma C., Kathuria S., Chowdhary A., Xu J. (2016). Diversity and origins of Indian multi-triazole resistant strains of *Aspergillus fumigatus*. Mycoses.

[75] Clinical and Laboratory Standards Institute (2008). Reference Method for Broth Dilution Antifungal Susceptibility Testing of Filamentous Fungi.

[76] Dunne K., Hagen F., Pomeroy N., Meis J.F., Rogers T.R. (2017). Intercountry transfer of triazole-resistant *Aspergillus fumigatus* on Plant Bulbs. Clin. Infect. Dis..

[77] Lamoth F. (2016). *Aspergillus fumigatus*-related species in clinical practice. Front. Microbiol..

[78] Tang Q., Tian S., Yu N., Zhang X., Jia X., Zhai H., Sun Q., Han L. (2016). Development and evaluation of a loop-mediated isothermal amplification method for rapid detection of *Aspergillus fumigatus*. J. Clin. Microbiol..

[79] Powers-Fletcher M.V., Hanson K.E. (2016). Molecular diagnostic testing for *Aspergillus*. J. Clin. Microbiol..

[80] Etienne K.A., Kano R., Balajee S.A. (2009). Development and validation of a microsphere-based luminex assay for rapid identification of clinically relevant *Aspergilli*. J. Clin. Microbiol..

[81] Bhimji A., Bhaskaran A., Singer L.G., Kumar D., Humar A., Pavan R., Lipton J., Kuruvilla J., Schuh A., Yee K. (2018). *Aspergillus* galactomannan detection in exhaled breath condensate compared to bronchoalveolar lavage fluid for the diagnosis of invasive aspergillosis in immunocompromised patients. Clin. Microbiol. Infect..

[82] Mello E., Posteraro B., Vella A., De Carolis E., Torelli R., D’Inzeo T., Verweij P.E., Sanguinetti M. (2017). Susceptibility testing of common and uncommon *Aspergillus* species against posaconazole and other mold-active antifungal azoles using the Sensititre method. Antimicrob. Agents Chemother..

[83] Hageskal G., Kristensen R., Fristad R.F., Skaar I. (2011). Emerging pathogen *Aspergillus calidoustus* colonizes water distribution systems. Med. Mycol..

[84] Egli A., Fuller J., Humar A., Lien D., Weinkauf J., Nador R., Kapasi A., Kumar D. (2012). Emergence of *Aspergillus calidoustus* infection in the era of post-transplantation azole prophylaxis. Transplantation.

[85] Springer J., White P.L., Hamilton S., Michel D., Barnes R.A., Einsele H., Löffler J. (2016). Comparison of performance characteristics of *Aspergillus* PCR in testing a range of blood-based samples in accordance with international methodological recommendations. J. Clin. Microbiol..

[86] Sun W., Wang K., Gao W., Su X., Qian Q., Lu X., Song Y., Guo Y., Shi Y. (2011). Evaluation of PCR on bronchoalveolar lavage fluid for diagnosis of invasive aspergillosis: A bivariate meta-analysis and systematic review. PLoS ONE.

[87] Reinwald M., Buchheidt D., Hummel M., Duerken M., Bertz H., Schwerdtfeger R., Reuter S., Kiehl M.G., Barreto-Miranda M., Hofmann W.-K. (2013). Diagnostic performance of an *Aspergillus*-specific nested PCR assay in cerebrospinal fluid samples of immunocompromised patients for detection of central nervous system aspergillosis. PLoS ONE.

[88] White P.L., Bretagne S., Klingspor L., Melchers W.J.G., McCulloch E., Schulz B., Finnstrom N., Mengoli C., Barnes R.A., Donnelly J.P. (2010). European *Aspergillus* PCR initiative *Aspergillus* PCR: One step closer to standardization. J. Clin. Microbiol..

[89] White P.L., Perry M.D., Loeffler J., Melchers W., Klingspor L., Bretagne S., McCulloch E., Cuenca-Estrella M., Finnstrom N., Donnelly J.P. (2010). European *Aspergillus* PCR initiative critical stages of extracting DNA from *Aspergillus fumigatus* in whole-blood specimens. J. Clin. Microbiol..

[90] Alanio A., Bretagne S. (2017). Challenges in microbiological diagnosis of invasive *Aspergillus* infections. F1000Research.

[91] White P.L., Parr C., Thornton C., Barnes R.A. (2013). Evaluation of real-time PCR, galactomannan enzyme-linked immunosorbent assay (ELISA), and a novel lateral-flow device for diagnosis of invasive aspergillosis. J. Clin. Microbiol..

[92] Aguado J.M., Vázquez L., Fernández-Ruiz M., Villaescusa T., Ruiz-Camps I., Barba P., Silva J.T., Batlle M., Solano C., Gallardo D. (2015). PCRAGA Study Group; Spanish Stem Cell Transplantation Group; Study Group of Medical Mycology of the Spanish Society of Clinical Microbiology and Infectious Diseases; Spanish Network for Research in Infectious Diseases. Serum galactomannan versus a combination of galactomannan and polymerase chain reaction-based *Aspergillus* DNA detection for early therapy of invasive aspergillosis in high-risk hematological patients: A randomized controlled trial. Clin. Infect. Dis..

[93] Hebart H., Klingspor L., Klingebiel T., Loeffler J., Tollemar J., Ljungman P., Wandt H., Schaefer-Eckart K., Dornbusch H.J., Meisner C. (2009). A prospective randomized controlled trial comparing PCR-based and empirical treatment with liposomal amphotericin B in patients after allo-SCT. Bone Marrow Transplant..

[94] De Pauw B., Walsh T.J., Donnelly J.P., Stevens D.A., Edwards J.E., Calandra T., Pappas P.G., Maertens J., Lortholary O., Kauffman C.A. (2008). Revised definitions of invasive fungal disease from the European Organization for Research and Treatment of Cancer/Invasive Fungal Infections Cooperative Group and the National Institute of Allergy and Infectious Diseases Mycoses Study Group (EORTC/MSG) consensus group. Clin. Infect. Dis..

